# Supplementary material for: CRISPR-Cas9 human gene replacement and phenomic characterization in Caenorhabditis elegans to understand the functional conservation of human genes and decipher variants of uncertain significance
Source: Dis Model Mech. 2018 Nov 26;11(12):dmm036517. doi: 10.1242/dmm.036517 (PMC6307914; doi:10.1242/dmm.036517)
Supplement: Supplementary information [file dmm-11-036517-s1.pdf]

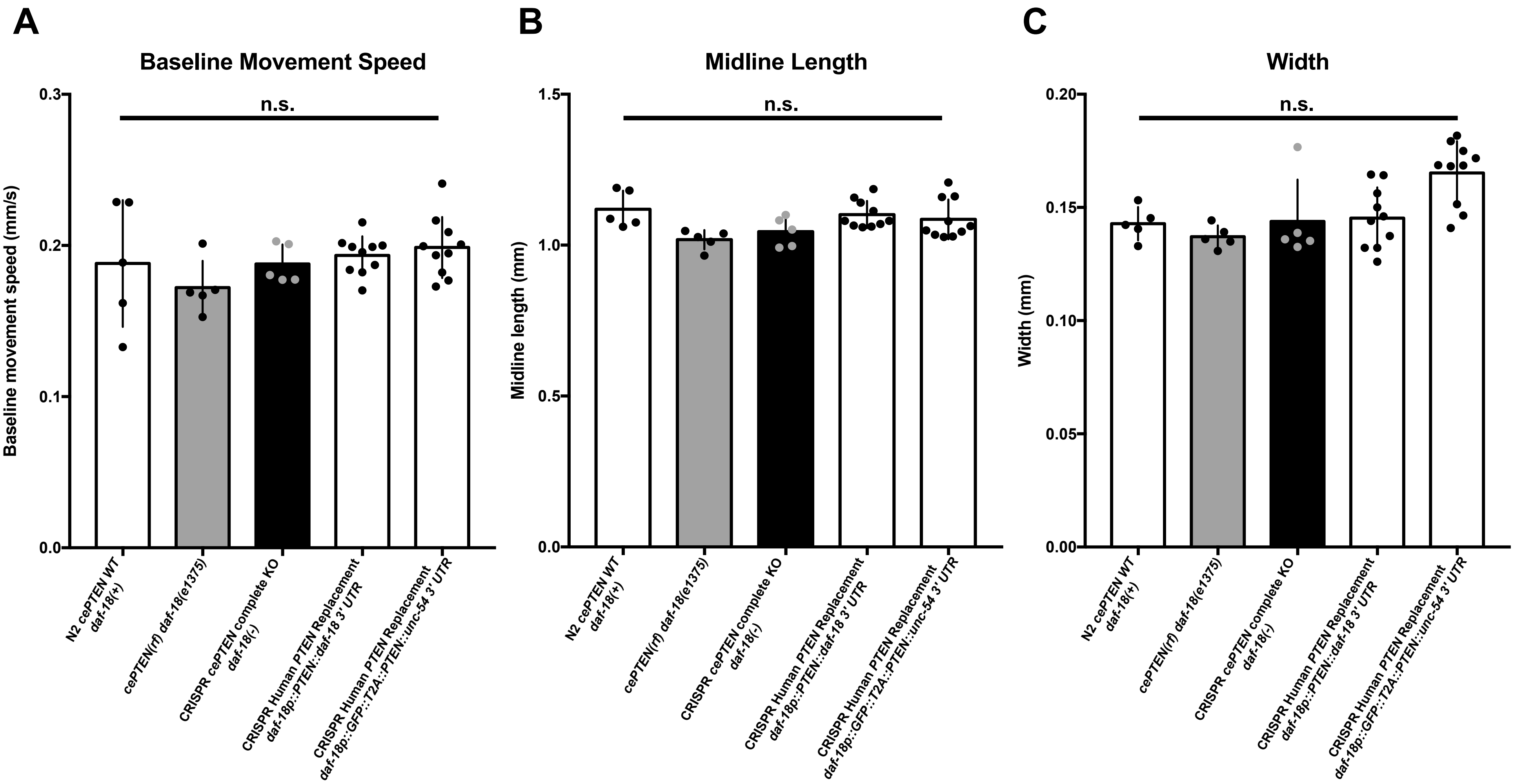

Figure S1 | Morphology and baseline locomotion are superficially normal in *daf-18* mutants and *PTEN* transgenic animals. A) Baseline movement speed, **B)** midline length, and **C)** width are not significantly different across genotypes. Circles represent plate replicates run on the same day. Error bars represent standard deviation using the number of plates as n (n = 5 or 10). n.s. not significant, one-way ANOVA and Tukey's post-hoc test.

Disease Models & Mechanisms • Supplementary information
